# Effects of light exposure during IVF: transcriptomic analysis of murine embryos and embryo-derived EVs

**DOI:** 10.3389/fimmu.2025.1429252

**Published:** 2025-02-20

**Authors:** Bence Nagy, Zoltán Bognár, Timea Judit Csabai, Nóra Fekete, Edit Irén Buzás, Árpád Ferenc Kovács, Júlia Szekeres-Barthó, Éva Pállinger

**Affiliations:** ^1^ Institute of Genetics, Cell- and Immunobiology, Semmelweis University, Budapest, Hungary; ^2^ Department of Medical Biology and Central Electron Microscopic Laboratory, Medical School, University of Pécs, Pécs, Hungary; ^3^ Szentágothai János Research Center, Pécs, Hungary; ^4^ National Laboratory on Human Reproduction, University of Pécs, Pécs, Hungary; ^5^ MTA - PTE Human Reproduction Research Group, Pécs, Hungary; ^6^ Tűzoltó Utcai Unit, Department of Pediatrics, Semmelweis University, Budapest, Hungary

**Keywords:** embryo, implantation, light exposure, extracellular vesicle, RNA-seq

## Abstract

**Introduction:**

Light exposure of embryos during assisted reproduction affects embryo quality and implantation capacity in a wavelength dependent manner. We investigated the molecular mechanism of these light-induced changes through the comparative analysis of gene expression and regulatory miRNA profile of murine embryos cultured in dark environment and those exposed to white- or red filtered light. miRNA sequencing was used to assess the role of embryo-derived extracellular vesicles in the endometrium-embryo dialogue.

**Methods:**

*In vitro* cultured mouse embryos at 3.5 days post coitum (dpc) were exposed to white or red filtered light. After 24 hours mRNA and miRNA content of the embryos as well as the miRNA content of embryo-derived extracellular vesicles were isolated and RNA-sequencing was performed. Differential expression analysis and functional enrichment analysis were used for evaluating the transcriptome results.

**Results:**

Light exposure caused transcriptomic changes in the embryos. White light upregulated apoptotic pathways, while red filtered light gave rise to the activation of regeneration pathways, including DNA repair mechanisms. Embryo-derived extracellular vesicles enclosed wavelength dependently unique miRNA cargos the target genes of which play a role in embryo implantation.

**Discussion:**

White light upregulates apoptotic pathways, at both the transcriptome and regulatory miRNAs levels. Red filtration partially counterbalances these negative effects by shifting the cellular processes towards regeneration, including DNA repair mechanisms. Extracellular vesicles of light exposed embryos play a role in blastocyst-decidua communication through the horizontal transfer of regulatory miRNAs. Our data prove that light exposure during *in vitro* fertilization modifies cell function that might affect the outcome of implantation.

## Graphical Abstract

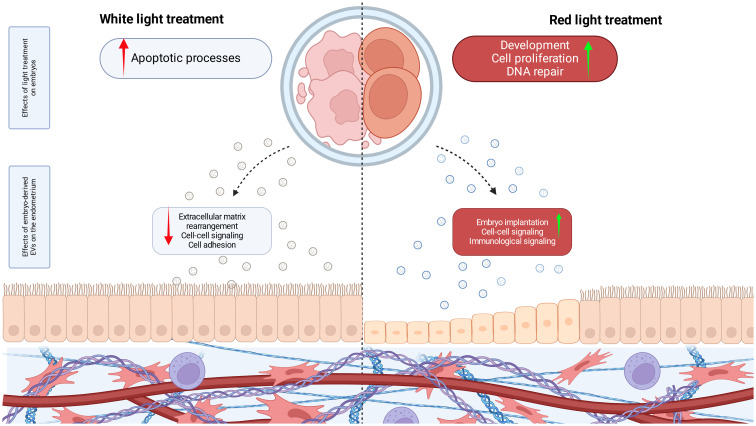

## 1 Introduction

The transition from the zygote to the blastocyst stage is characterized by rapid cell division and the activation of several genes that regulate embryo differentiation. Therefore, it is accompanied by a characteristic transcriptome. However, these biological processes make the embryos extremely vulnerable to environmental changes (1, 2). During assisted reproduction, human embryos are exposed to light, which is a completely new environmental effect, because during natural conception, embryos develop in the dark. Light can trigger the production of reactive oxygen radicals, alter the gene expression profile and thus, affect the cell differentiation and division processes (3–5). Despite all this, evidence regarding the implantation capacity of light-exposed embryos is sparse. Therefore, we investigated the effects of light exposure, on *in vitro* cultured mouse embryos.

Extracellular vesicles (EVs) are membrane-covered particles containing complex molecular cargoes. Their production is phylogenetically conserved, and recent evidence suggests that they play an essential role in interkingdom communication (6). There is also evidence that EVs may have a role in human reproduction (7). To the best of our knowledge, embryo-derived signals, including EVs, are essential for endometrial maturation and influence the success of implantation (8–11). In our previous studies we have also demonstrated: 1) the presence of EVs in embryo culture fluids (12), and 2) the light-induced change in the EV composition of embryo culture fluids (13).

We have previously demonstrated a wavelength-dependent relationship between light exposure and embryo development and implantation in cultured mouse embryos. White light exposure significantly reduced the implantation rate of mouse embryos compared to either those cultured in a dark environment or the ones exposed to red filtered light. White light caused embryonic cell damage, including DNA fragmentation and modified the embryo-derived extracellular vesicle (EV) profile. White light-exposed embryos released higher amounts of double stranded nucleic acid containing (propidium iodide positive) EVs. Since we have previously demonstrated that adherence of mouse embryo-derived EVs to splenocytes induces the IL-10 production in CD8+ cells, we monitored the effects of EVs secreted from light-exposed mouse embryos. Contrary to the systemic effects of EVs from embryos cultured in dark environment or those exposed to red filtered light, EVs released after white light exposure do not induce IL-10 production of splenocytes. We concluded that the harmful effect on implantation capacity could be related to the wavelength of the light. White light treatment induced DNA fragmentation and apoptosis of *in vitro* cultured mouse embryos resulting in implantation failure. These effects are partially counterbalanced by using a red filter (13).

The aim of our study was to investigate the molecular mechanism of light-induced cell processes through the comparative analysis of gene expression and regulatory miRNA profile of murine embryos cultured in dark environment or those exposed to white-or red filtered light. The secondary goal was to investigate the EV-mediated communication between the *in vitro* fertilized embryos and the endometrium. To answer these questions, we examined the miRNA composition of EVs isolated from the culture medium of the *in vitro* fertilized, light treated mouse embryos.

## 2 Methods

### 2.1 Embryo retrieval, culture and light treatment of embryos

Embryo retrieval, culturing and light exposure of *in vitro* cultured embryos were performed as described earlier. In brief, 48 hours after injection of 5 IU FSH (Merional, IBSA Pharma, Switzerland) into 12-week-old CD1 female mice (Charles River, Germany), the animals were treated with 5 IU LH (Choragon, Ferring, Hungary) and mated directly with CD1 males. After 2 days, two-cell stage embryos were collected from the oviducts and cultured in 0.4% BSA containing KSOM medium (Millipore, England) under mineral oil (37°C, 5% CO2). The number of embryos varied between 10 and 14 in drops. The medium was changed every 24 hours. Morphologically intact two-cell stage embryos at 2.5 dpc were transferred to M2 medium (Millipore England), exposed to light and cultured for a further 24 hours. The procedure was repeated at the 4-cell stage. The light intensity of the compact lamp used for treatment was measured with a digital luminometer (Hold Peak - HP 881B). To compare the wavelength-dependent effects of the light, the intensity was set at 1130 lx in each case (13).

Three treatment groups were formed:

1. Control embryos: 2 × 50 minutes (min) incubation at room temperature in the dark (n = 30).
2. Embryos subjected to white light (1130 lx) for 2 × 50 min (n = 30).
3. Embryos subjected to red filtered light (1130 lx) for 2 × 50 min (n = 30).

Twenty-four hours after light exposure, the embryos were scored for developmental stage. (**Table 1**) Embryos with morphological changes associated with degeneration and cell division arrest were considered non-viable and were excluded from further investigations. Light stressed and control embryos (day 3.5) were frozen in RNAlater Stabilization Solution (ThermoFisher, USA) to stabilize and protect cellular RNA for next generation sequencing. Samples were stored at -20°C until use.

**Table 1:** Development and implantation capacity of embryos after light stress (brightness of red and white light are both 1130 lx).

Embryo development				
Developmental stage	post coital day (dpc)	White light stressed No of embryos (%)	Red light stressed No of embryos (%)	Control No of embryos (%)
morulas + early blastocysts + non-viable	3.5	293 (100%)	203 (100%)	360 (100%)
non-expanded blastocysts	4.5	39 (13.3%)	47 (23.2%)	16 (4.4%)
expanded blastocysts + hatching blastocysts	4.5	201 (68.6%)	118 (58.1)	304 (84.5%)
non-viable	4.5	53 (18.1%)	38 (18.7%)	40 (11.1%)
Implantation				
Implanted/transferred	3.5	31/156 (19.9%)*	21/56 (37.5%)*	66/115 (57.4%)

*p<0.05 indicating a significant difference compared to the control.

Conditioned embryo-culture media were pooled and frozen at -80°C until RNA isolation.

### 2.2 Library preparation for transcriptome and miRNA analysis from mouse embryos

For RNA sequencing, total RNA was isolated from murine embryos using the RNeasy MiniKit (Qiagen, Venlo, Netherlands) according to the recommendations of the manufacturer. The RNA integrity was evaluated with Agilent Tapestation system (Agilent, Santa Clara, CA, USA) and samples with RIN values ≥7 were further processed. For library preparation, multiplex Small RNA Library Prep Kit (Illumina, Inc., UK) was used according to the manufacturer’s instructions. The sequencing was performed on an Illumina MiSeq instrument and the paired-end read value was > 20 million/sample.

### 2.3 EV isolation and characterization

A spin column-based method was used for the isolation of total RNA from EVs in embryo-culture media. EVs were isolated from conditioned embryo culture media and non-conditioned medium controls by exoEasy Midi Kit (Qiagen, Venlo, Netherlands), then using miRNeasy Mini Kit (Qiagen, Venlo, Netherlands) to isolate RNA from EVs. The standard protocol is described in the exoRNeasy Serum/Plasma Handbook (Qiagen, Venlo, Netherlands) (**Supplementary Figure 1**).

### 2.4 Isolation of miRNA from embryo-derived EVs, library preparation and next generation sequencing

To reveal the detailed miRNA content of embryo-derived EVs, next-generation miRNA sequencing was applied. Conditioned media of cultured embryos were collected and pooled. Ten conditioned medium samples were pooled in each treatment group and three biological replicates were analyzed. RNA content of embryo-derived EVs was isolated with exoRNeasy Serum/Plasma Midi Kit (Qiagen, Venlo, Netherlands). The RNA integrity was evaluated with Agilent Tapestation system (Agilent, Santa Clara, CA, USA) and samples with RIN values ≥7 were further processed. For library preparation, multiplex Small RNA Library Prep Kit (Illumina, Inc., UK) was used according to the manufacturer’s instructions. The sequencing was performed on an Illumina MiSeq instrument and the paired-end read value was > 20 million/sample.

### 2.5 mRNA analysis

Quality control (QC) pipeline for transcriptome analysis included a first QC check on raw sequence data provided by FASTQC software (14). For identification, transcripts were fit to the GRCm39 as a reference genome, and pseudoalignment was generated by the Kallisto v0.46.1 software (15), with the paired-end option, a bootstrap sampling on the reads was repeated 100 times, with the default 42 seeds. The estimated standard deviation of the fragment length was 20. The genome alignment resulted in pseudobam files, which were converted into BAM files with the Samtools v1.17 software (16). Summarizing the abundances of the transcripts and the identification of their genes were performed by the featureCounts program (17) within the Rsubread v2.16 package. DeSeq2 v1.42 software (18) was used for differential expression analysis. Gene ontology enrichment analysis was completed with ClusterProfiler v4.10 software using the Gene Ontology “Biological Processes” database (DOA:2023.03.20.) (19, 20), and pathway differences with a p<0.05 were considered significant. GeneTonic v2.6 R package was used for the visualization of the results (21) (**Supplementary Figure 2**).

### 2.6 miRNA analysis

Quality check (QC) pipeline for miRNA analysis included quality assurance of miRNA sequences by FASTQC software (14). Prior to downstream analysis, reads were trimmed to 18-35 bp in length by Seed 2.0 software (22). For identification, transcripts were aligned to the GRCm39 murine reference genome by the Bowtie2 v2.5.1 program (23), and data were converted into BAM files with the Samtools v1.17 software. Calculation of miRNAs’ abundances and identification of their targets featureCounts software within the Rsubread v2.16 package was used. For functional interpretation of miRNA-target interaction networks, we used the miRNet 2.0 platform (24) with the “Expression table” analysis option. Normalization of data was achieved by trimmed mean of M-values (TMM) with a Tagwise dispersion estimation. Statistical analysis was fulfilled with the built-in edgeR software (25) and p<0.05 was accepted as significant. Target genes of miRNAs were identified by miRTarBase v8.0 program (26). Pathway analysis of the target genes was performed using the Gene Ontology “Biological Processes” (DOA:2023.10.30.) and the “Reactome” (DOA:2023.10.30.) databases. p<0.05 was accepted as statistically significant (**Supplementary Figure 3**).

## 3 Results

### 3.1 Light-induced transcriptional activity of embryonic cells

Our previous results show that light-stressed embryos reduce their implantation capacity (13). In attempt to identify the underlying mechanisms, first we investigated the changes in the transcriptome of light exposed embryos. mRNA sequencing identified 10 589 genes expressed in murine embryos, with exclusive expression of 1144, 1367 and 1295 genes in white light-treated, red light-treated and control embryos, respectively.

As a result of the differential gene expression analysis (compared to the untreated control sample), we identified 156 genes that showed significant expression changes in both light-treated groups. However, the gene expression pattern also showed wavelength dependence: 528 genes were expressed exclusively in the embryos illuminated with white light and we found 486 genes expressed only in the red filtered light-exposed embryos.

**Figure 1:**
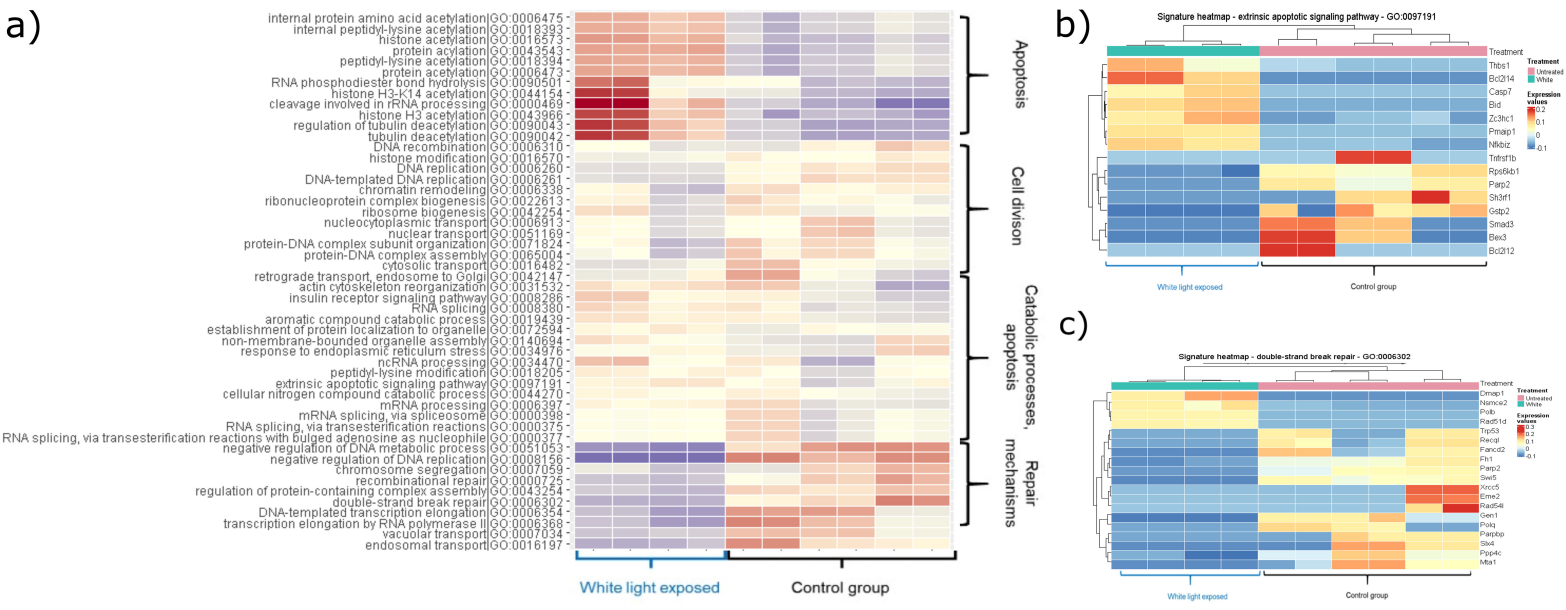
Heatmaps showing the significantly changed pathways within the white light-treated group. The colouring correlates with the Z-values, with red indicating an increase and blue indicating a decrease. **(A)** Heatmap showing upregulated and downregulated pathways induced by white light treatment. **(B)** The heatmap shows the up- and down-regulated genes of the extrinsic apoptotic signalling pathway (GO:0097191) compared to the control group (adj. P-value: 0.035995). **(C)** The heatmap shows the up- and down-regulated genes of the double-strand break repair mechanism (GO:0006302) compared to the control group (adj. P-value: 0.016647493).

The enrichment analysis of the unique gene expression profiles of white light-treated group proved the upregulation of apoptotic pathways (GO:0097191 (extrinsic apoptotic signaling pathway) adj. P-val. = 0.035995), and downregulation of DNA repair mechanisms (GO:0006302 (double-strand break repair) adj. P-val. = 0.016547493; GO:0000725 (recombinational repair) adj. P-val. = 0.035995398) (**Figure 1**).

**Figure 2:**
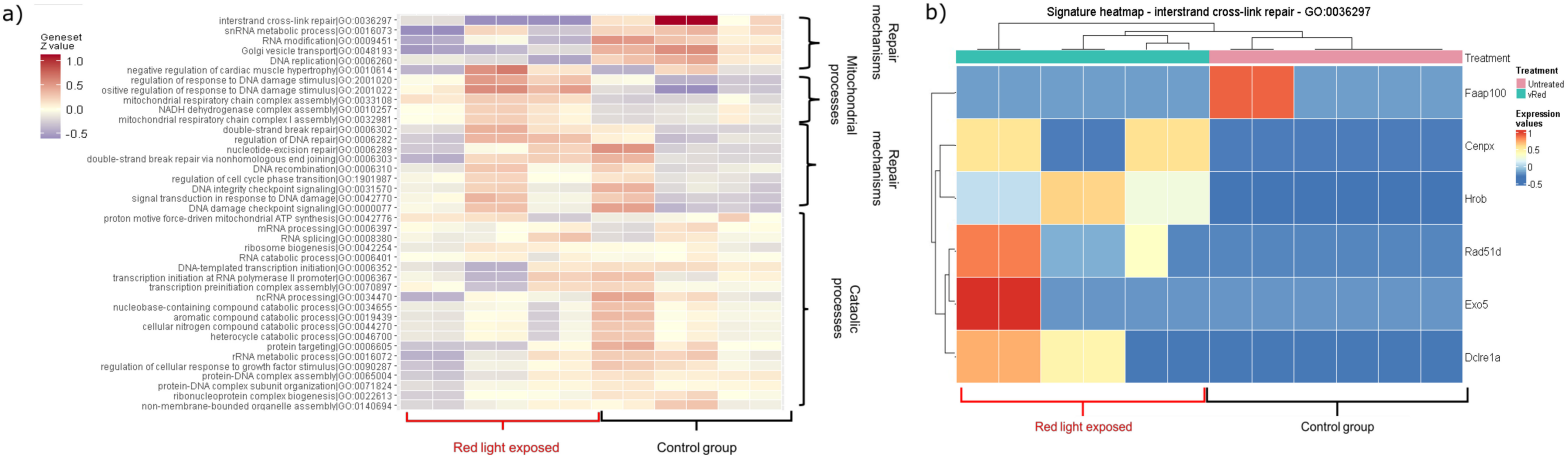
Heatmaps showing the significantly changed pathways within the red filtered light-treated group. The coloring correlates with the Z-values, with red indicating an increase and blue indicating a decrease. **(A)** Heatmap showing up- and downregulated pathways induced by red-filtered light treatment. **(B)** The heatmap shows the up- and down-regulated genes of the interstrand cross-link repair mechanism (GO: 0036297) compared to the control group (adj. P-value: 0.006994897).

In the red light-exposed group, the apoptotic pathways did not change significantly, but DNA repair mechanisms, cellular processes were elevated and many pathways involved in mitochondrial functions were downregulated. These results confirm our previous findings 1) increased apoptosis in embryos exposed to white light, 2) provide explanation for the effects of red filtered light by showing upregulated repair mechanisms in samples from red light-exposed embryos (**Figure 2**) (**Supplementary Table 1**: significantly changed genes after light exposure; **Supplementary Table 2**: enrichment analysis of the significantly changed genes after light exposure).

### 3.2 Light-induced miRNA pattern of embryonic cells

Light exposure induced wavelength-dependent changes in the levels of three miRNAs (mmu-mir-184-3p, mmu-mir-351-5p, mmu-mir-5119). The levels of these miRNAs decreased in response to white light, whereas they increased in response to red light. Although we did not find miRNAs that exclusively changed after the white light treatment of embryos, exposure to red filtered light was associated with the significant increase of mmu-mir-30b-5p and mmu-mir-5124a and the significant downregulation of mmu-mir-423-3p. Identification of miRNAs’ targets resulted in 68 and 117 genes in the white and the red light treated groups, respectively.

**Figure 3:**
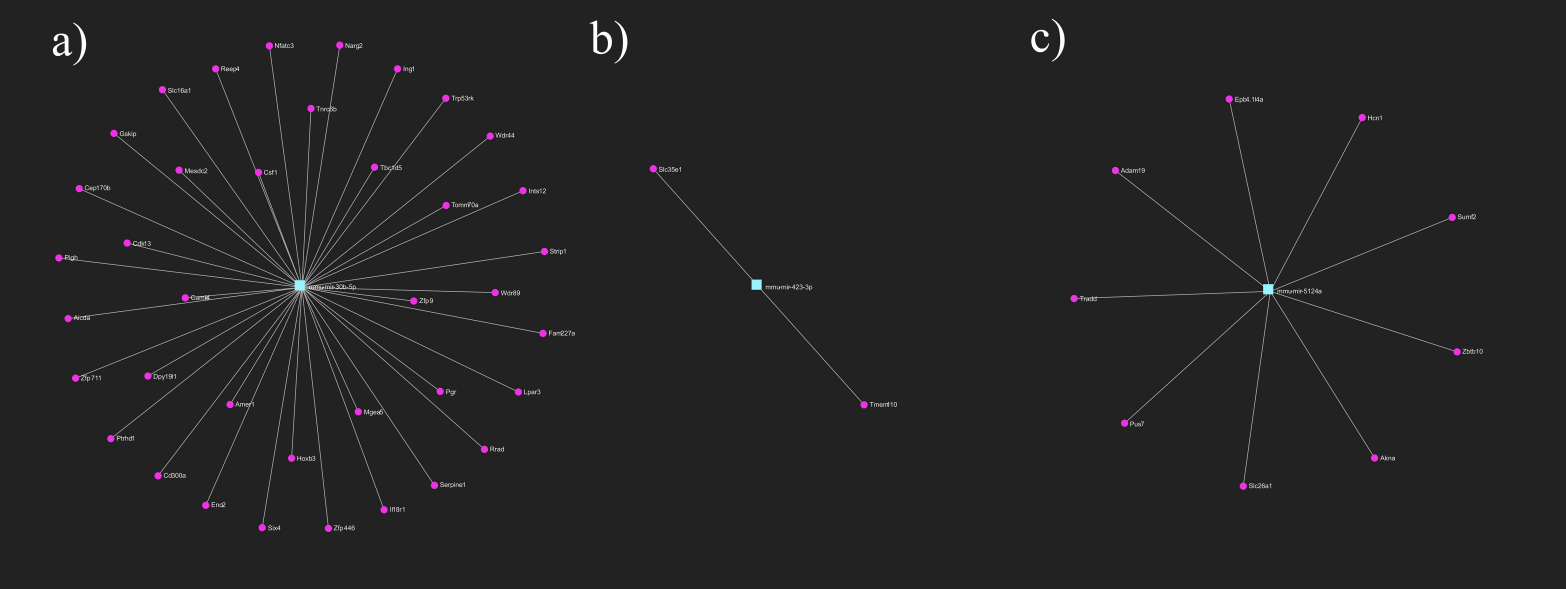
Primary gene targets of differentially expressed miRNAs after light exposure. Target genes of **(A)** mmu-mir-30b-5p **(B)** mmu-mir-423-3p **(C)** mmu-mir-5124a. Turquoise rectangles represent the miRNAs, magenta circles represent their target genes. miRNA-target gene network has been constructed using miRNet 2.0 online platform.

Since miRNAs are involved in the regulation of various genes, we used pathway enrichment analysis to give meaning to high-throughput miRNA data. The relative abundance of genes pertinent to specific pathways was measured through statistical methods, and associated functional pathways were retrieved from the Reactome and the GO: BP databases. Calculation of adjusted p-values allowed to exclude false positive results, so we had a chance to interpret the real differences between groups (27, 28). Pathway analysis of target genes of mmu-mir-351-5p, including Stat1 (Signal Transducer And Activator Of Transcription 1) and Hif1an (Hypoxia Inducible Factor 1 Subunit Alpha Inhibitor) have a significant role in several pathways such as cellular responses to hypoxia (adj. P-val. = 0.00193), and immunological signaling (Interleukin-6 signaling, adj. P-val. = 0.0193; Interferon gamma signaling, adj. P-val. = 0.0318). Therefore, white light-induced decrease of mmu-mir-351-5p may affect the biological behavior of embryo (embryo quality) and later may determine the implantation outcome. Although exposure to red light is also related to the mmu-mir-351-5p interactome, the pathway analysis confirms targets involved in the regulation of the cell cycle (G1 phase, adj. P-val. = 0.0242). Pathway analysis also verified the connection between the red light dependent miRNA profile and the cellular metabolic (glucose metabolism, adj. P-val. = 0.0368) and developmental processes (immune system development, adj. P-val. = 0.00984). Predicted target genes of miR-5119 are involved in RNA splicing and RNA metabolisms (GO) and in apoptotic processes (Reactome DOA) (**Figure 3**).

The miRNA changes detected only in red light illuminated embryos mainly affect the metabolic activity of the cells. miR-423 is a cellular ATP regulator targeting genes involved in mitochondrial energy metabolism (29), while miR-30b is involved in mitochondrial protein transport, cell differentiation and glucose metabolism.

The differences in wavelength-dependent implantation competence proven in *in vivo* experiments are reflected in the miRNA population differences. That is, the intracellular miRNAs influence embryo quality and may predict implantation outcome.

### 3.3 Light exposure induces unique miRNA cargo in embryo-derived EVs

**Figure 4:**
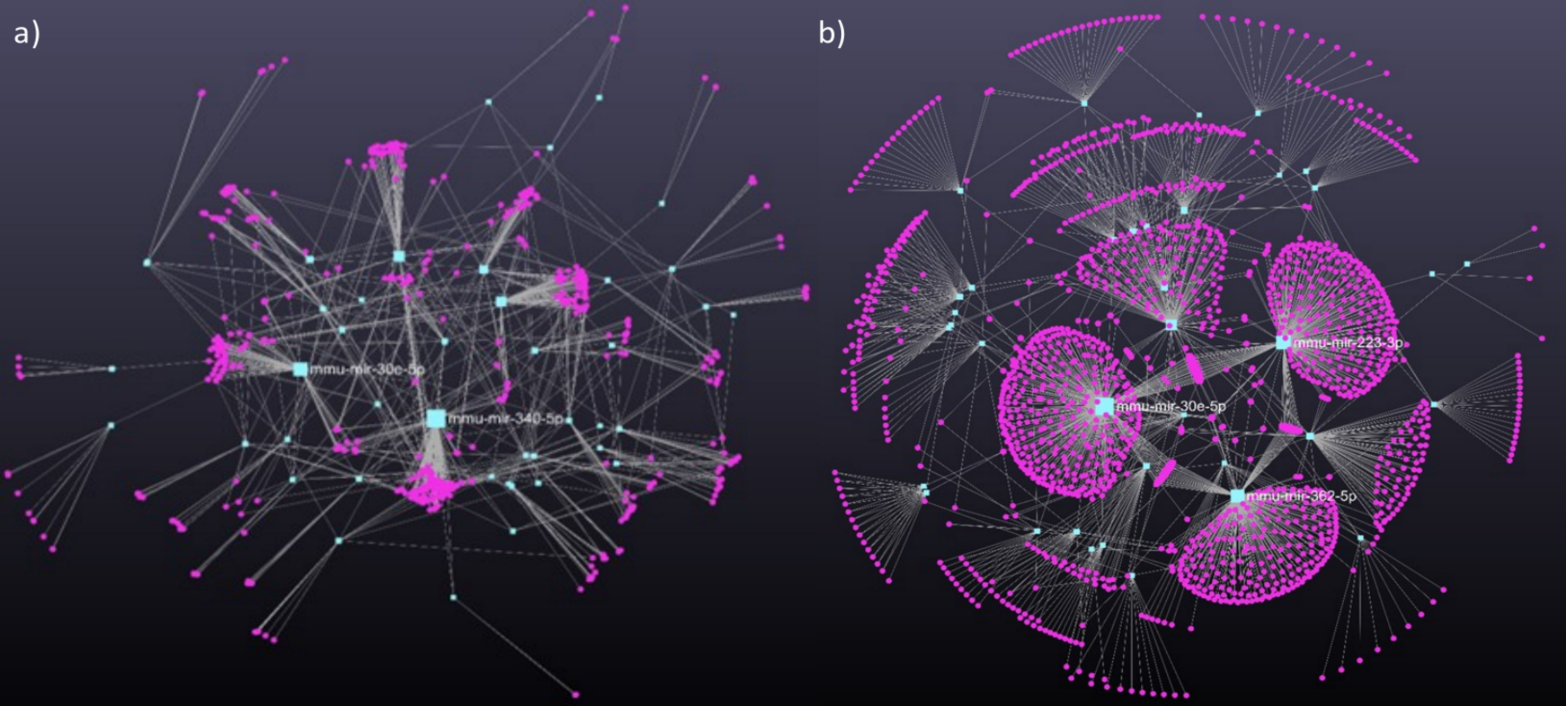
The interactome of significantly changed miRNAs from embryo-derived EVs **(A)** filtered red light treatment **(B)** white light treatment. Turquoise rectangles represent the miRNAs, magenta circles represent their target genes. miRNA-target gene network has been constructed using miRNet 2.0 online platform.

miRNA-seq of EVs in conditioned media resulted in 160, 99 and 83 miRNAs in the untreated control group, in the white light illuminated and in the red light-exposed groups, respectively. According to the comparative analysis, 61 miRNAs were present in all samples. White light treatment induced 13 unique miRNAs, while red filtered light exposure resulted in only 5 exclusive miRNAs. Sixty-seven miRNAs could be detected in both treated groups (**Figure 4**).

Light exposure induced miRNA secretion of pre-implantation embryos is associated with implantation outcome. Therefore, embryo-derived miRNAs may serve as potential biomarkers in the screening process during ART.

### 3.4 The effects of white light treatment on miRNA profile of embryo-derived EVs

Differential expression analysis showed significant change in the case of 54 miRNAs compared to the control group, 30 of which were downregulated and 24 were upregulated. Downregulated miRNAs target 1610 genes that are associated with cell-cell signaling (adj. P-val. = 0.00000403), cell-matrix adhesion (adj. P-val. = 0.000011), cell development (adj. P-val. = 0.000035) or tissue remodeling (adj. P-val. = 0.00327). We highlight the mmu-mir-223-3p that affects genes regulating Rho GTPase functions (adj. P-val.0.00234), VEGF, EGFR and PDGF signalization (adj. P-val. = 0.00686; adj. P-val. = 0.000676; adj. P-val. = 0.000879) and also influence immunological pathways, including DAP12 (adj. P-val. = 0.00325) and Fc epsilon receptor (FCERI) signaling (adj. P-val. = 0.014). From the point of view of implantation, mmu-mir-29a-3p is also an important regulator, because its target genes are involved in extracellular matrix organization (adj. P-val. = 1.72e-10). Upregulated miRNAs target 226 genes but the pathway analysis did not demonstrate significant involvement, except in the case of mmu-mir-532-5p whose many target genes are involved in cell adhesion (adj. P-val. = 0.03). In summary, exposure to white light changes the miRNA cargo of embryo-derived EVs.

### 3.5 Effects of red light treatment on miRNA profile of embryo-derived EVs

We detected 62 miRNAs (24 upregulated and 38 downregulated) in the conditioned media of red light treated mouse embryos, the expression of which significantly differed from the control group. Target gene analysis identified 787 genes associated with upregulated miRNAs and 1698 genes that connect to downregulated miRNAs. Downregulated miRNAs regulate genes that play a role in cellular metabolism, cell-cell adhesion, tissue and embryo development, cell cycle, tissue remodeling, and immune signaling. Functional enrichment analysis revealed that cell adhesion, cell migration, and signalization pathways were significantly affected. Particularly important are the effects of mmu-mir-503-5p (adj. P-val. = 0.00000356) and mmu-mir-210-3p (adj. P-val. = 0.008), which target reproductive processes, of the inflammation associated mmu-mir-375-3p (adj. P-val. = 0.0668) and of mmu-mir-29a-3p (adj. P-val. = 0.0000156), which are involved in extracellular matrix reorganization.

In summary, light treatment affects the miRNA cargo of embryonic-derived EVs in a wavelength-dependent manner. In contrast to white light treatment, the miRNA pattern induced by red light exposure enables regeneration processes that support the implantation capacity of embryos compared to their white light-treated counterparts.

## 4 Discussion

Successful implantation requires intensive bidirectional feto-maternal communication at the molecular level. The endometrium is sensitive to embryo-derived mediators that can influence endometrial receptivity and implantation. There is evidence that the signals emitted by poor-quality embryos prevent the development of the essential endometrial microenvironment (30) including the transformation of endometrial stromal cells (ESC) into decidual stromal cells (DSC) (31). In addition to soluble molecules and mediators, extracellular vesicles are also involved in the endometrium-embryo dialogue. EVs conveying complex messages affect the function of target cells, thus the cargo of embryo-derived EVs can affect the function of endometrial epithelial and immune cells, and modulate the biosensor function of the endometrium (32, 33).

During assisted reproduction, the *in vitro* cultured embryos are exposed to environmental stress that affects the molecular composition of embryo-derived messages. Soluble mediators and EVs derived from poor-quality embryos arrest the development of the proper endometrial microenvironment and cause implantation failure (34). Therefore, we examined the miRNA cargo pattern in EVs in conditioned media of *in vitro* cultured control or light- exposed mouse embryos. We aimed to clarify, whether messages carried by embryo-derived EVs might play a role in the altered implantation rate of light-treated embryos.

Visible light is an important environmental stress factor during assisted reproduction. Although it does not have sufficient energy to break the chemical bonds of organic macromolecules, it causes functional changes in cells, primarily through alteration of mitochondrial processes. Wavelength dependent photon absorption of mitochondrial cytochrome c oxidase accelerates the electron transport chain and increases ATP synthesis (35–37). We have previously demonstrated that white light exposure of the embryo induces implantation failure and suggested that microscopic analysis of the embryo is not sufficient to predict the implantation outcome. We also showed that the white light-induced negative effects can be partially counterbalanced by using a red filter, and that the biological effects induced by visible light depend on the wavelength rather than the intensity of the light (13). In the present study, we identified the complex molecular mechanism of light-induced cell processes by comparative transcriptome and regulatory miRNA profile analysis of murine embryos. We also characterized the miRNA cargo of EVs produced by light –treated mouse embryos.

Highly regulated specific gene expression profile controls blastocyst activation, which is a major determining factor for implantation. Single-cell RNA sequencing of peri-implantation mouse and human embryos demonstrated downregulated pathways of protein metabolism, energy production, mitochondrion organization, implantation-associated genes (like *FGF13* and *RBP7*), DNA repair mechanisms and 18 S ribosomal RNA m6A methylation in embryos that fail to attach. On the other hand, upregulated translational elongation genes (such as *RPS28* and *RPS29*) were detected (38, 39).

In our study, transcriptome analysis showed wavelength dependent significant differences in light-treated mouse embryos. White light-induced activation of extrinsic apoptotic signaling pathways with parallel downregulation of DNA repair mechanisms explain the implantation failure that was detected in our *in vivo* studies (13). It is consistent with the red light-induced upregulation of DNA repair mechanisms which explains the better implantation capacity of these embryos (13).

Transcriptome analysis is not complete without the simultaneous investigation of regulatory mechanisms. Therefore, we also examined the light exposure-induced miRNA patterns in mouse embryos. miRNAs control gene expression in a sequence-specific manner, by regulating the stability of mRNAs in nucleoli, regulating alternative splicing and directly guiding transcriptional gene activation (40). miRNA pattern, including the expression levels of mmu-mir-184-3p, mmu-mir-351-5p and mmu-mir-5119 changed in opposite directions; they were downregulated in white light treated and upregulated in red light exposed embryos. The target genes of these miRNAs are important in cell death processes and cell survival, although most of the miRNA data have become known in relation to cancers (41–43).

mir-184 is directly involved in the regulation of many cellular processes, including apoptosis and epigenetic regulation. It also exerts its effect by regulating the levels of other miRNAs.

miR-184 negatively regulates miR-205, the participant of miR-200 family, and through this pathway, it affects the transcriptional repressors of E-cadherin, ZEB1 and ZEB2 transcription factors (44, 45).

In the context of our study, miR-184-associated cell cycle regulation may explain our *in vivo* results. Overexpression of miR-184 in red light-treated embryos may have been associated with increased proliferation and cell cycle progression, similar to cancer cells.

This coincides with the downregulation of miR-184 after white light exposure. Higher expression level of miR-351 also has an impact on cell cycle via the regulation of lamin B1 expression and mitochondrial function (42). Target genes of miR-5119 are involved in RNA splicing and RNA metabolisms and in apoptotic processes. The miRNA changes associated with red light (miR-423 miR-30b) primarily affect the metabolic activity of cells, including the regulation of ATP synthesis and mitochondrial energy metabolism (29). miR-30b is also involved in mitochondrial protein transport processes, cell differentiation and glucose metabolism (46).

In summary, the wavelength-dependent transcriptomic changes are associated with cell cycle regulation and apoptotic pathways, which explain the impaired implantation activity of light exposed embryos.

Analysis of the light exposure-induced alterations in the transcriptome and in the regulatory miRNA patterns confirms the wavelength-dependent responses of embryonic cells to light stress. These results are consistent with our previous data showing increased DNA fragmentation in white light-induced embryos. mRNA and miRNA expressions also explain our *in vivo* results showing reduced implantation capacity of white light exposed embryos.

Several studies suggest the role of *miRNAs* in embryo implantation. Early studies used *in vitro* models, for identifying miRNAs involved in decidualization (47, 48). The interactome of these miRNAs involves cytokines, extracellular matrix enzymes, growth and transcriptional factors. Pre-implantation miRNAs such as mir-661 that correlates with implantation failure play a role in blastocyst-decidua communication and are associated with implantation outcome (49),. Investigation of miRNA content of conditioned media of *in vitro* cultured embryos is a non-invasive method that can increase the success rate of IVF (50–52).

EVs are potent mediators of signaling between the endometrium and embryo. Endometrial EVs have an impact on embryo development and implantation. For example, EVs secreted by endometrial cells of patients with recurrent implantation failure delay blastocyst differentiation (53). On the other hand, co-culturing of embryos with endometrium-derived mesenchymal stem cell-derived EVs may improve the competence of aged oocytes (54). The embryo - endometrium crosstalk is bidirectional: embryo-derived EVs customize the endometrium and thereby control implantation (55, 56). In this study, we also investigated the EV-mediated messages of control and light exposed embryos. Comparison of the miRNA content of light-exposed embryos and embryo-derived EVs resulted in miRNA patterns different from the controls. This difference supports that the embryos launch a well-defined miRNA EV cargo that is capable of transmitting specific messages. Although the mechanism of miRNA sorting into EVs is not yet fully understood, the wavelength dependence of miRNA patterns clearly confirms that the miRNA composition of embryo-derived EVs is not the consequence of a random selection, but is a strictly controlled, conscious message that determines the fate of implantation (57). Differential expression analysis showed significant changes in the miRNA patterns of embryo-derived EVs after light exposure compared to the control group. We also identified wavelength dependent unique miRNA cargo in embryo-derived EVs. Exposure to white and red filtered lights induced the exclusive expression of 13 and 5 miRNAs, respectively. Based on the analysis of the target genes of the detected miRNAs, we found that the significant miRNA changes affect cell-matrix interaction, cell differentiation and tissue transformation. We highlighted the mmu-mir-223-3p because it affects genes that regulate among others the signalization of growth factors (VEGF, EGFR, PDGF) and different immunological pathways. We concluded that light induced miRNA messages affect among others the signalization of growth factors (mir-223-3p), immunological pathways (mir-223-3p, mir-375-3p), extracellular matrix organization (mir-29a-3p), cell adhesion (mir-532-5p), and cellular metabolism, tissue and embryo development (mir-503-5p). All pathways are important factors for embryo implantation, so our data presented here prove that light exposure induced changes in cell function affect the outcome of implantation.

## 5 Conclusion

During IVF and ICSI, the embryos are exposed to environmental stress. White light exposure of murine embryos results in reduced implantation capacity. The underlying mechanisms of this phenomenon include altered transcriptional activity regulated by miRNAs and consequently, an altered cargo of embryo-derived EVs. Taken together; these data suggest that light protection of the embryos covering the white light sources with a red filter causes well-defined alterations in the mRNA and miRNA content both in the embryos and in embryo-derived EVs. These changes are consistent with our previous results showing an improvement in the implantation rate of red light-exposed embryos compared to white-treated counterparts at the same illumination level.

## Data Availability

The datasets presented in this study can be found in online repositories. The names of the repository/repositories and accession number(s) can be found below: GSE266335 (GEO).
