# Supplementary material for: Effects of light exposure during IVF: transcriptomic analysis of murine embryos and embryo-derived EVs
Source: Front Immunol. 2025 Feb 20;16:1429252. doi: 10.3389/fimmu.2025.1429252 (PMC11882875; doi:10.3389/fimmu.2025.1429252)
Supplement: Supplementary file 1 [file DataSheet1.docx]

Supplementary Material

**Protective effects of red filtered light during IVF: transcriptomic analysis of murine embryos and embryo-derived EVs**

Bence Nagy^1*^, Zoltán Bognár^2,3,4,5^, Timea Judit Csabai^2,3,4,5^, Nóra Fekete^1^, Edit Irén Buzás^1^, Árpád Ferenc Kovács^6^, Júlia Szekeres-Barthó^2,3,4,5^ ^†^, Éva Pállinger^1*^ ^†^

***Correspondance:** Bence Nagy: nagy.bencep94@gmail.com

**1 Supplementary data**

The datasets presented in this study can be found in online repositories. The names of the repository/repositories and accession number(s) can be found below: <https://www.ncbi.nlm.nih.gov/geo/query/acc.cgi?acc=GSE266335>

Accession number: GSE266335

**2 Supplementary Figures and Tables**

**2.1 Supplementary figures**

**Supplementary figure 1.**Exosome purification and total RNA isolation from embryo culture media. Modified figure of Qiagen exoRNeasy Serum/Plasma Kit.

**Supplementary figure 2.** Workflow of mRNA analysis

**Supplementary figure 3.** Workflow of miRNA analysis

**2.2 Supplementary Tables**

**Supplementary table 1.** The results of the differential expression analysis showing the upregulated genes in the treatment groups after light exposure.

**Supplementary table 2.** The results of the functional enrichment analysis of the significantly changed genes in the different treatment groups after light exposure
