# Supplementary figures and images for: Effects of light exposure during IVF: transcriptomic analysis of murine embryos and embryo-derived EVs
Source: Front Immunol. 2025 Feb 20;16:1429252. doi: 10.3389/fimmu.2025.1429252 (PMC11882875; doi:10.3389/fimmu.2025.1429252)

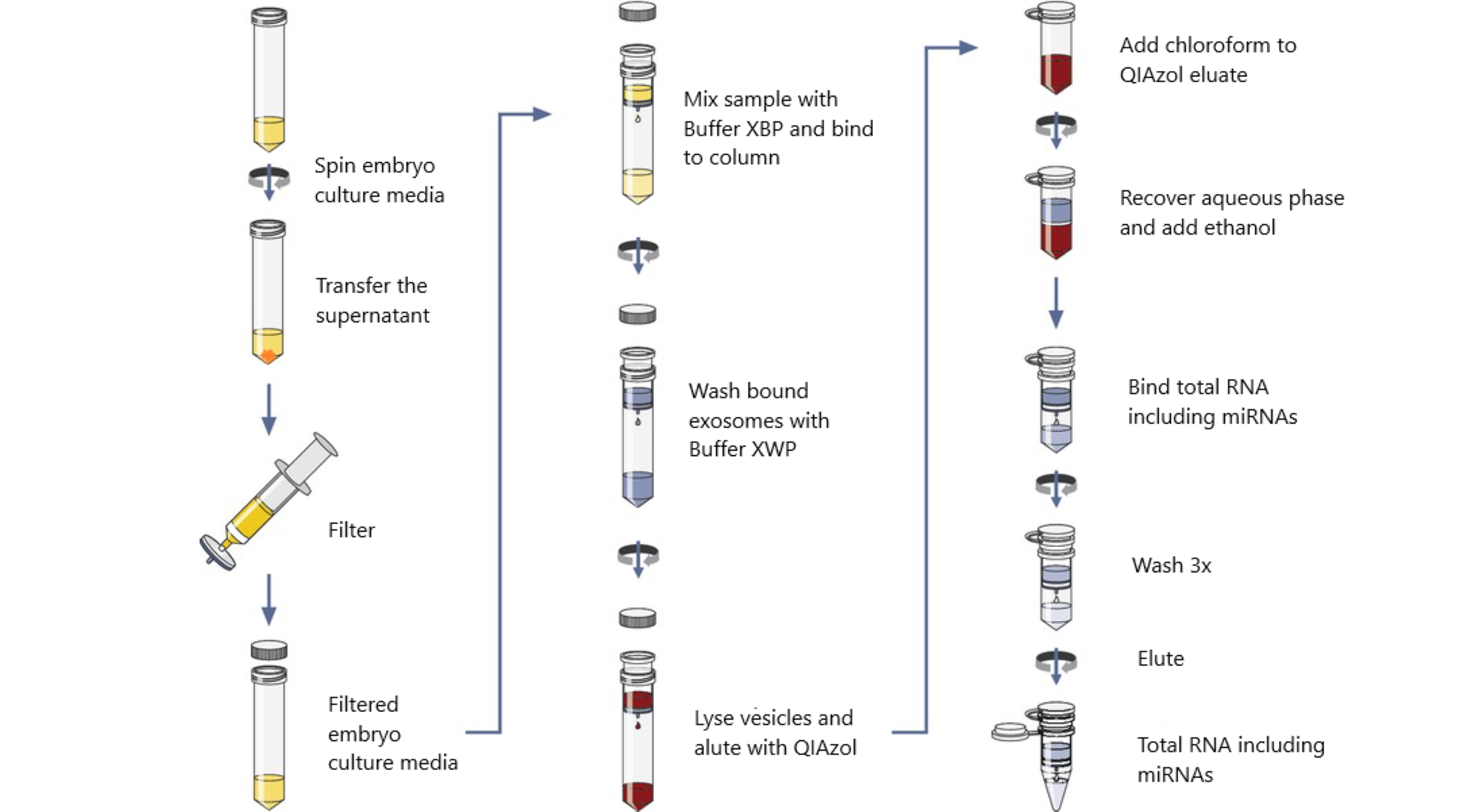

Supplement: Supplementary file 2 [file Image1.tif]

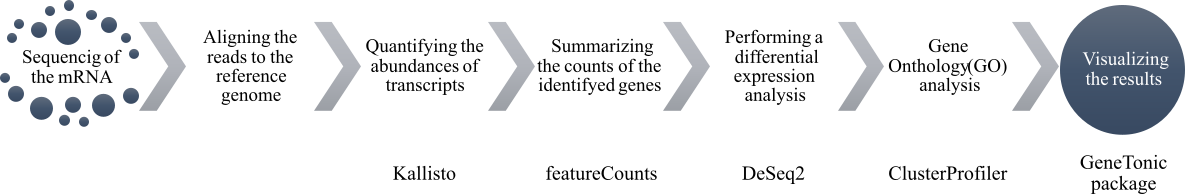

Supplement: Supplementary file 3 [file Image2.tiff]

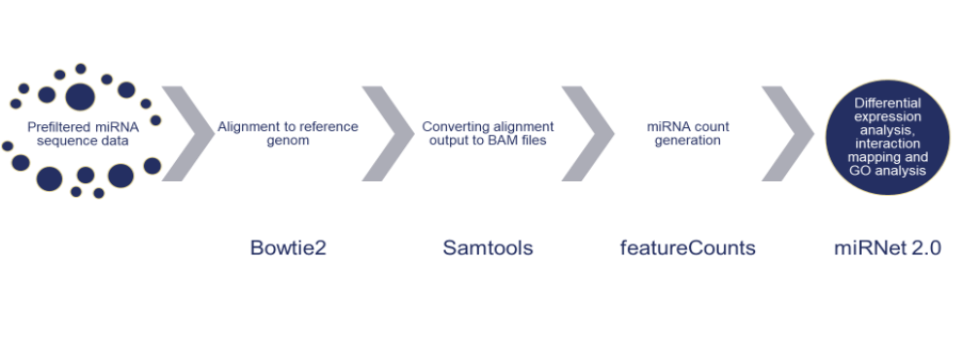

Supplement: Supplementary file 4 [file Image3.tiff]
